# Promoting mental health and wellbeing in schools: the impact of yoga on young people's relaxation and stress levels

**DOI:** 10.3389/fpsyg.2023.1083028

**Published:** 2023-05-17

**Authors:** Ingunn Hagen, Solbjørg Skjelstad, Usha Sidana Nayar

**Affiliations:** ^1^Department of Psychology, Norwegian University of Science and Technology (NTNU), Trondheim, Norway; ^2^Stabilisati Counseling Service, Psychological Counseling, Yoga and Meditation Guidance, Levanger, Norway; ^3^Department of Psychology, Tata Institute of Social Sciences (TISS), Mumbai, India

**Keywords:** yogic relaxation, yoga in school, young people, mental health and wellbeing, yoga for stress, yoga and sleep

## Abstract

This study aimed to examine students' experiences with yoga interventions in school. The findings revealed that practicing yoga made young people more aware of their need to relax and positively impacted their mental health and wellbeing. We explored the emphasis on relaxation among our study participants and how relaxation is related to other aspects, such as their experience of stress and sleep habits. This article is based on qualitative data gathered from teenagers in Norway who participated in the Norwegian part of the European research project “Hippocampus: Promoting Mental Health and Wellbeing among Young People through Yoga.” The project introduced yoga to young people in schools with an emphasis on those who were disadvantaged, including those with mental health issues and other challenges related to their background. Data were collected from nine individual, semi-structured interviews and 133 logs collected in the spring of 2019.^1^ The major themes identified through the interviews and log material included becoming more relaxed and aware of the need to unwind. Thus, the focus on relaxation is based on the importance of the participants assigned to this theme. The study results suggest that yoga enhanced the students' awareness and skills and empowered them to make healthier lifestyle choices.^2^ We viewed the importance of relaxation from a salutogenic perspective, focusing on the factors that contribute to good health in contrast to a pathogenic perspective, where curing diseases is the goal.

## 1. Introduction

In contemporary society, young people face numerous challenges related to their mental health and wellbeing, a situation that has been exacerbated by the COVID-19 pandemic. A recent study reported that mental health among adolescents and young people has been declining over the past three decades, based on a national, cross-sectional study in Norway (Krokstad et al., 2022). This study also found a significant increase in depression and anxiety among adolescents and young adults, as well as a decrease in life satisfaction over the last decade. Nearly 44% of the young girls in this study reported being stressed and having depressive thoughts. The poor mental health of these young people was interpreted to be due to their living conditions and daily behaviors, such as a sedentary lifestyle in front of screens and the great impact of social media. In addition to the effects related to recent technological innovations, young people are faced with concerns related to climate change and threats to democracy and social justice. However, the most important stressor was increased performance pressure in school. The authors blame the neoliberal ideology that places an emphasis on competition, which has had an increasing impact over the last few years.

Another study found similar tendencies for stress and mental health challenges in young people, attributed to extensive performance pressure in school and social expectations of success in other ascpects of life (Eriksen et al., 2017).^3^ Adolescence can be a challenging and stressful period in life because it is a time when several external and internal changes occur. Internally, there are changes in the brain, body, and hormones (Guyer et al., 2016). Externally, there are extensive social and cultural changes, such as the recent developments related to technology use. Similar to their global counterparts, most youngsters in Norway are heavy social media users, and such platforms have been designed for addiction (Rice et al., 2018, 2020; Smahel et al., 2020). Due to their constant use of social media and being available to their friends 24/7, young people are often sleep-deprived (Nayar et al., 2012). Their lack of sleep is also a result of their stress levels (cf. Khalsa, 2004; Khalsa and Butzer, 2016). Lack of sleep can lower positive emotions and, over time, be a risk factor for young people's mental health and wellbeing (Saksvik-Lehouillier et al., 2020).

This study focused on how introducing yoga to young people can facilitate their wellbeing and improve their mental health. In this article, we will discuss how yoga makes teenagers feel relaxed rather than stressed and sleep-deprived. Our study was part of the European research project “Hippocampus: Promoting Mental Health and Wellbeing among Young People through Yoga.^4^”

While our study is limited to the results from Norway, we have presented the Hippocampus Project below, which shares our common aim and research design. We have also provided an overview of the main quantitative results to provide background information.

The Hippocampus Project aimed to improve the wellbeing of young people by giving them yogic tools to cope with the challenges and stress of their everyday lives. More precisely, the objective was to investigate how practicing yoga impacted their wellbeing, stress, and sleep patterns. The project focused on the issues facing disadvantaged youth and offered yoga classes to them and other teenagers in school settings. The initiative introduced young people to an 8-week-long yoga intervention with weekly yoga sessions.

We found the salutogenic model of health introduced by Aaron Antonovsky in 1979 to be a highly relevant perspective when examining a yoga intervention. This model presupposes that the origins of good health are not necessarily the same as those of poor health or pathogenesis (Antonovsky, 1996). Thus, the emphasis is on factors that promote health and ease rather than disease. In the present study, we attempted to understand how yoga facilitates relaxation among youth and how being relaxed may counteract stress and improve sleep. Yoga is a suitable tool since it offers a whole-person approach, which conforms to the salutogenic perspective described by Antonovsky (1996). International research on salutogenetic mechanisms has confirmed that people with a high “sense of coherence” (SOC) about their world have better health. SOC consists of the following three elements: comprehensibility, manageability, and meaningfulness (Antonovsky, 1996; Mittelmark and Bauer, 2022). The salutogenic perspective, similar to yoga, is suitable for integrative medicine or health care.

## 2. Background and perspectives

### 2.1. Young people in Norway: a generation with performance pressure?

In Norway, young adults are increasingly experiencing stress, pressure, and mental health challenges (Krokstad et al., 2022). Bakken et al. (2019) found that stress levels varied; however, youngsters typically experienced burdens at school and the pressure to look fit and be successful in terms conveyed through social media. Norwegian youth were among the most intense social media users in Europe, especially through their mobile phones (Smahel et al., 2020). Over a third of the young adults in Norway spend more than 4 h a day in front of a screen, in addition to the screen time they may have at school (Bakken, 2019). General lack of physical activity and sleep problems among the youth reinforce their problems (Hysing et al., 2015; Haug et al., 2020). The discourses on young people are often characterized by what they lack, what is wrong, and problems related to growing up with performance pressure, stress, and fear related to social comparisons.

Research has shown that young people attribute their stress levels and mental health challenges to the demands put on them in school (Eriksen et al., 2017). Expectations from parents, especially middle-class parents, tended to exacerbate the pressure to perform well in school, forcing young people to measure their self-worth based on their achievements (Eriksen, 2020). Parents differed in how explicitly they expressed their expectations, but many expressed concerns about their children's future security. For young people, this added to the pressure they already experienced regarding their academic performance, and they also expressed worries concerning their future educational and job opportunities.^5^ Bakken (2019) found that most Norwegian adolescents were content with their lives and experienced wellbeing and good mental health.^6^ Despite this, the author identified numerous negative stress factors, such as higher drug consumption and violence, increased loneliness, reduced optimism about the future, more screen time and use of social media, and lower levels of wellbeing at school.

In general, young refugees in Norway face some of the same stressors and mental health challenges as other young Norwegians. In addition, they have various disadvantages related to not knowing the Norwegian language and culture and stress related to being in transition and having been exposed to trauma, thus developing varied degrees of posttraumatic stress disorder (PTSD). A recent study showed that young refugees often faced mental health issues, and half of the unaccompanied refugee minors had symptoms of PTSD when they arrived in Norway (Sund et al., 2019). Furthermore, they had high levels of psychological distress, and the symptoms of elevated stress levels often persisted over time (Jakobsen et al., 2017).

### 2.2. Mental health and wellbeing: how can yoga contribute?

Since we are discussing the experiences related to the Hippocampus Research Yoga Intervention, it will be useful to describe a few relevant principles of yoga, which is known as an ancient, diverse, and vast science. One of the most frequently quoted definitions of yoga, “yogah chittivrtti nirodhah,” is the second sutra by the well-known codifier of yoga, Maharishi Patanjali, in his *Yoga Sutras*. This sutra has numerous interpretations and translations, such as “Yoga is the cessation of mental modifications of the mind” (Yogendra and Hansaji, 2011, p. 6) or “Yoga is the stilling of mental turbulence” (White, 2007, p. 16). Central yogic principles include the following eight limbs of yoga in Patanjali's *Yoga Sutras*: Yamas (social restrictions), Niyamas (internal disciplines), Asana (physical postures), Pranayama (control of prana/life energy through breath), Pratyahara (withdrawal of the senses), Dharana (concentration), Dhyana (meditation), and Samadhi (union or integration) (see Hagen, 2018).

How can yoga contribute to mental health and wellbeing? Yoga can be defined as a process of conscious evolution and 4-fold awareness by increasing people's awareness of their body, mind, and emotions and by developing meta-awareness, which starts by realizing one's lack of awareness (Gitananda, 1981). Moreover, yoga is claimed to improve all aspects of health, including mental, physical, emotional, social, and spiritual (Bhavanani, 2014). Psychological health benefits often include increased somatic awareness, improved mood, and subjective wellbeing. Practicing yoga can boost self-acceptance, self-actualization, and social adjustment and thus reduce anxiety and depression. Cognitive functions may be enhanced in terms of improved attention, concentration, memory, and learning (Bhavanani, 2014). Yoga can be defined as a “skill in action,” ideally without expectations (Gitananda, 1981), which may increase balance and resilience. Physically, yoga can induce balance in the autonomic nervous system by activating the parasympathetic part more than the stress-induced sympathetic part. Thus, this physical effect may help reduce stress and mental health challenges for the younger generation.

However, what is stress, and how can yoga contribute to coping with and counteracting it? The concept of stress is often credited to Hans Selye, who introduced the term in medicine as a “non-specific response of the body to any demand” (see Tan and Yip, 2018). Selye described stress as underpinning the non-specific symptoms of illness and linked how the body coped with stress to the hypothalamic–pituitary axis. He identified three stages in the stress reaction, namely the alarm reaction, the resistance phase, and the exhaustion phase. Stress is often associated with the first stage and can be defined as “an exaggerated response to a change in the external or internal environment” (Bhavanani, 2016).^7^ Stress is a set of responses to a stimulus that disturbs the mental or physiological equilibrium. The perception of a stressful event depends on the individual and can trigger the fight, flight, or freeze response, thereby causing certain hormones, such as adrenaline and cortisol, to be produced in the body.^8^ Long-term stress or an overactive sympathetic response is not ideal for physical or mental health. Some level of sympathetic activation is needed to perform well in life, but young people also need to rest and digest, as they do in the parasympathetic mode. The yogic ideal is to achieve a balance between the sympathetic and parasympathetic modes (Udupa and Sathyaprabha, 2018).

Yogic relaxation differs from other forms, in that it is aimed more actively (Gitananda, 1981). Relaxation is important for contemporary human beings, including young people, because the modern lifestyle forces most people to live under constant stress. Stress refers to being in a state of over activation where the sympathetic part of the autonomic nervous system is constantly triggered. However, life needs to consist of both activity and rest, tension and non-tension; therefore, parasympathetic activation and sympathetic activation are required.^9^ Compared to ordinary relaxation, yogic relaxation is not only about physical relaxation but also about developing mind control over our nervous and glandular systems, which are important to controlling the human body (Gitananda, 1981 p. 107). True relaxation means being free of worries and cultivating a relaxed attitude in one's daily activities. By being relaxed, one can continue ongoing activities without becoming exhausted.

### 2.3. Research on yoga in school and stress: what about relaxation?

School settings have been the preferred arena for interventions when offering yoga courses to teenagers (Khalsa et al., 2012; Noggle et al., 2012; Serwacki and Cook-Cottone, 2012; Khalsa, 2013). This was also the case with the Hippocampus Project, which makes sense since much of the stress experienced by youth is school-related. In an introduction to an anthology about yoga in school, Khalsa explains the following:

*The physical postures/exercises, breath regulation, and relaxation techniques in yoga may be particularly relevant to facilitating contemplative practice in a young population that has high levels of psychophysiological energy and arousal and who is not accustomed to sitting in stillness for extended periods. There is therefore a compelling rationale for the implementation of yoga in school settings* (Khalsa, 2015: xii).

It is important to reduce stress among young people in school settings and to understand the causes of stress and how yoga might help reduce stress (see Frank et al., 2014; Riley and Park, 2015; Dariotis et al., 2016; Park et al., 2021). Batista and Dantas (2002) proposed that yoga can be one of the most powerful agents for controlling stress. In their recent review of the impact of yoga on stress among healthy adults, Wang and Szabo (2020) found that most yoga types had positive effects on stress reduction, at least in healthy populations. Similarly, a review study of children and the benefits of yoga concluded that practicing yoga improved children's ability to cope with stress and reduced their experience of stress and anxiety (Nanthakumar, 2018). Furthermore, Butzer et al. (2017, p. 1) concluded, “Students had particularly positive opinions regarding the beneficial effects of yoga on stress, sleep, and relaxation.” A British study found that yoga helped teenagers deal with the daily stress of school, thus helping them feel better about themselves and become more capable of coping with school requirements (Morgan, 2014). Interviewees in Morgan's study also found it easier to be themselves or the person they wanted to be when practicing yoga, which helped reduce their stress levels. Another study on yoga in school (Conboy et al., 2013) reported the following psychological benefits for students: “many cited stress reduction; many used yoga to manage negative emotions; and some propagated more optimism” (p. 171). Similarly, Frank et al. (2020) suggested that yoga (sports) reduced the perception of stress and accompanying mental health symptoms, such as anxiety and depression.

Moreover, practicing yoga has been found to have a positive effect on posttraumatic stress disorder (PTSD) and trauma-related conditions, including anxiety, depression, and sleep disorders (Balasubramaniam et al., 2013; van der Kolk et al., 2014; Canadian Agency for Drugs and Technologies in Health, 2015; Olsen, 2020). Contact with one's body through yoga and facilitating the ability to self-regulate tended to contribute to traumatic healing experiences (Nguyen-Feng et al., 2019). People with PTSD often encounter problems falling asleep, but yoga was found to improve insomnia among individuals with PTSD (Jindani et al., 2015). While it may improve mental health, wellness, and symptoms related to PTSD, in serious cases of PTSD, yoga should be adjunctive to other forms of treatment.

Thus, research on yoga in school has mainly focused on how practicing it can improve the way young people cope with stress, which is rather crucial. In this study, however, we were more concerned with how young people can learn to relax and counteract stress in their everyday lives, as a better balance between rest and activation is beneficial for their physical wellbeing and mental health. In line with the salutogenic perspective, highlighting the health benefits of relaxation can be an important addition as a solution-oriented approach rather than a problem-focused perspective to research yoga for coping with or reducing stress.

## 3. The hippocampus project: reflections on intervention and methodology

### 3.1. How to recruit schools and conduct a yoga intervention?

The Norwegian research design was shaped by the European five-country Hippocampus Project that we were part of.^10^ The goal was to improve the wellbeing of young people, including disadvantaged youth, by offering them weekly yoga sessions for 8 weeks.

We invited four different schools to participate in the Norwegian Hippocampus Project. The 8-week yoga course was first introduced to students and teachers at a high school that often collaborates with our university (HS1) and an international school (IS; offering an International Baccalaureate or high school diploma) during the fall of 2018. In 2019, we continued recruiting teenagers and teachers at a secondary school (SS). Since we wanted to include disadvantaged youth, we also recruited participants in “adult learning programs” for migrants and refugees, who attended two classes at another high school (HS2) and an introductory class at HS1. Thus, we offered yoga to young refugees from two different school program levels: one group of newly arrived refugees of high school age learning primary school curriculum at HS2, and another group participating in an introductory school program at HS1 focusing on a lower-level curriculum and being more skilled at speaking and writing in Norwegian.

This yoga program was developed by UK-based Charlotta Martinus (Please refer to Teenyoga.com), one of the UK Hippocampus partners. A total of 7 certified yoga teachers from Norway were recruited and trained to deliver the yoga program based on the manual describing the yoga program for young people (please refer to Hippocampusproject.eu).^11^ The core values for the Hippocampus Yoga Intervention were collaboration, compassion, and empowerment. Each week was supposed to build on the previous week so that the poses were repeated and values were reaffirmed, thereby providing the young people with a yoga “starter toolkit” for developing life skills (see Table 1). The yoga teachers were also requested to promote two of the eight limbs of yoga highlighted by Patanjali in the *Yoga Sutra*s, Yamas (social restrictions), and Niyamas (internal disciplines), which facilitates good relationships and a greater peace of mind. Other limbs mentioned earlier, such as asana (posture), pranayama (breath control), and meditation, were all integrated into the yoga classes.

**Table 1:** Themes and practice goals for the 8-week yoga intervention.

**Week**	**Theme**	**Practice goal**
One	Nourishing	What is yoga, busting any myths, introducing the basics of asanas and breathing.
Two	Building focus	Square breathing. The notion of focus. Particularly, how we can improve our attention span through our movements and breath.
Three	Building trust	Trust in the asanas and in relaxation.
Four	Compassion with self and others	Checking in with their body and how it feels, listening to themselves.
Five	Optimism	Creativity, fun, joy, partner postures, and pinnacle postures.
Six	Metta meditation or cultivating loving-kindness	Breathing–Ujjayi breath. Partner postures.
Seven	Radical self-care	Adapt practices to fit the mood and temperament.
Eight	Self-practice	Teaching each other to encourage home practice.

The 8-week yoga course intervention in Norway included 201 students and 49 teachers in four different schools. After the intervention, we were interested in how the participants perceived yoga and its potential benefits.

Below are the research questions that we will attempt to answer in this study:

1. What was the experience of the young students at the Hippocampus Yoga Intervention offered in Norwegian schools?
2. Did the students feel that the yoga course impacted their mental health and wellbeing; their view on stress; and their sleep patterns, and, if so, in what way?

### 3.2. Reflections on methodology and data: focus on logs and qualitative interviews

The Hippocampus Project combined quantitative and qualitative data. The survey had a pre- and post-intervention design. The questionnaire was sent to all the young people who participated in the yoga classes and to the teachers who had their own yoga classes after school. The qualitative part consisted of semi-structured, qualitative individual interviews (with disadvantaged youth), group interviews (with teachers), logs (from young people in Norway only), and reflection notes (from yoga teachers). Interviews were conducted with young people who had experienced some form of disadvantaged circumstance in their lives. In the logs, all the students could write down their experiences in their own words.

For the qualitative interviews, we followed the guidelines from the project agreement to recruit disadvantaged participants. In the Hippocampus Project, being disadvantaged was defined as being a young person between the ages of 13 and 25 years in a situation with one or more of the following challenges: mental health, physical health, psychosocial, and socioeconomic. Since few wished to be identified as “disadvantaged,” we implemented an open invitation to participate in interviews in the information letter and consent form. We also explained to the teachers that we were interested in young people in situations of disadvantage.

Our sample of interviewees was the result of these combined channels of effort and distribution of project information, as well as the efforts of students who volunteered to participate. Thus, the qualitative interviews comprised nine students with certain disadvantages, such as Norwegian students with mental health issues (e.g., anxiety or ADHD) and/or students with a refugee background. The Norwegian interviewees were identified as having mental health issues or other challenges, either by themselves or through their teachers' knowledge about the students' difficulties. The refugee students qualified as disadvantaged owing to their limited knowledge of the Norwegian language and culture and carrying traumatic memories of war and the loss of close family members. Some of them had been living in Norway as underage refugees without any family and with various degrees of PTSD. The refugee interviewees had different origins and situations: two well-integrated girls from Syria and three others who recently arrived in Norway: an Afghan boy, a Kurdish boy, and a Somali girl. Refugee informants have experienced various degrees of conflict and war in their lives, in addition to various challenges that are often part of fleeing from their country of origin. Table 2 presents the details of the participants recruited for the qualitative interviews. Fictional names have been used to safeguard their anonymity.

**Table 2:** Participant's details.

**Name (fictive)**	**Gender**	**Age span, years**	**Country of origin**	**Disadvantage**	**Grade level**
Ayla	Girl	17–20	Syria	Refugee/trauma	2nd year HS2
Barbara	Girl	17–20	Syria	Refugee/trauma	2nd year HS2
Cole	Boy	13–16	Norway/Lithuania	Difficult in class	9th grade SI
David	Boy	13–16	Norway	ADHD	8th grade SS
Emily	Girl	13–16	Norway	Anxiety	8th grade SS
Fay	Girl	17–20	Norway	ADHD	3rd year HS1
Hugir	Boy	17–20	Kurdistan	Refugee/trauma	Introductory program HS1
Idil	Girl	13–16	Somalia	Refugee/trauma	Introductory program HS1
Javid	Boy	17–20	Afghanistan	Refugee/trauma	Introductory program HS1

In Norway, we also collected written reflections from students at the four schools participating in the project and gathered 133 logbooks or logs. The logs were mostly written at school and contained the students' experiences during yoga practice and the effects they experienced. The students were asked to share their impressions and reflections based on these experiences.

The qualitative interviews were performed by Solbjørg Skjelstad and took place in quiet rooms in various school settings. Each interview lasted approximately 30 m. Only the interviewer, interviewee, and mother language assistant (MLA) were present when a translation was needed. The interviews were semi-structured based on an interview guide developed by Nick Kearny, which was discussed with others on the Hippocampus team. In line with Kvale, we regarded the purpose of the qualitative research interview as obtaining a “description and interpretation of themes in the subjects' lived world,” with a continuum between the description and the interpretation (Kvale, 1996, p. 187). Interviews in Norway were conducted flexibly with the guide as a checklist while the interviewer was constantly probing and seeking clarification, as well as potential interpretations, from the informants. The interview guide can be found in the Appendix.

The qualitative data analysis conducted in Norway was mainly thematic, based on the national data, and involved cooperation with other countries' project participants during the initial stages. The initial coding was discussed in Skype meetings where all five countries followed an initial coding scheme developed by Kearny. In this joint analysis process, we identified actualized themes according to wellbeing at school and with family and friends, stress, and sleep based on the interview guide. In Norway, we performed a thematic analysis, identifying the themes and patterns in the data, which was the point of departure for our interpretation, reflection, and reporting of the data (see Braun and Clarke, 2006, 2014; Braun et al., 2022). The starting point for establishing the themes was our research interest, as formulated in the research questions. More precisely, the thematic analysis consisted of several carefully conducted reviews of the nine interviews while taking notes according to the three layers of interpretation (descriptive, conceptual, and theoretical). This culminated in a list of interesting and relevant themes and patterns in the data. Thematic analysis of the qualitative interview data was performed manually by Skjelstad and was informed by discussions with co-authors Hagen and Nayar.^12^ A similar process was used for the log material by focusing on the main themes.

## 4. Results

### 4.1. Qualitative data: potential for a deeper understanding?

The joint survey result with all participants in the 8-week yoga course showed reduced perceived stress, fewer sleep issues, and improved overall wellbeing (please refer to Hippocampusproject.eu). These findings from all five countries were statistically significant, with moderate effect sizes for the changes. However, in the Norwegian survey, effect sizes were smaller, and no statistically significant differences between T1 and T2 were found [While 413 young people in the overall Hippocampus survey responded to the baseline dataset (T1), 339 young people responded to the follow-up (T2). Due to various factors of attrition, the matched dataset reached 260. The corresponding numbers for the Norwegian survey with young people were 133 (T1), 87 (T2), and 40 for the matched dataset].

Consequently, we had to rely on the qualitative data to deepen our understanding of how the participants interacted with the Hippocampus Yoga Intervention. We viewed this focus as useful since qualitative methodology has a unique logic and value through access to people's subjective experiences (Hagen, 1993). Additionally, we agree with Butzer et al. (2015) statement that qualitative research can provide an in-depth perspective on the impact of yoga interventions in schools.

An important advantage of the qualitative approach was that the informants could express their yoga experiences in their own words. We have presented the results from the qualitative sample of the Norwegian Hippocampus Project, the nine interviews with disadvantaged youth, and logs from 133 informants, below. For qualitative interviews, quantity is often sacrificed for the ability to go into depth in the interview conversation and the analysis. However, it adds to our numbers that we have 133 logs, which include statements from both the students in situations of disadvantage and their classmates. In our presentation of the results, we will first indicate the main themes identified in the interviews. Then, we will pay attention to the theme of relaxation, which was highly central in the interviews and logs. Finally, we will address how relaxation is related to wellbeing, stress, and sleep.

### 4.2. Qualitative research results: relaxation as a dominant theme

We present one of the central themes from the Norwegian qualitative individual interviews with young people. Here, we have chosen to focus on one overarching theme that was among the most dominant ones in the data, both in the interviews and especially in the log material:

“*Yoga as recognition of the need for relaxation*.^13^”

There were also other central themes, such as the following:

I. “*Yoga for regulation of sleep habits”*
II. “*Stress regulation and coping (with life situations)”*
III. “*Yoga's benefits for refugee trauma”*
IV. “*Yoga for emotional management”*
V. “*Enhanced attention, clarity, and focus”*
VI. “*Belonging.”*

We will address the other themes when we mention them in relation to the theme of relaxation.

In our results, we have focused on the most dominant theme of our study, namely, how yoga tends to impact the young informants' experiences of being relaxed, as well as making them more aware of their need to relax. We have further clarified how being relaxed tended to impact the participants' sleep habits and stress levels. All findings were based on Norwegian qualitative interviews and logs. The theme of relaxation was especially prominent in the logs, where approximately two-thirds of the responses referred to how yoga contributed to the informants becoming more relaxed. Our informants, both in the interviews and logs, also used words such as “becoming calm and peaceful with yoga.” This notion of calm was also one of the key themes identified in the joint analysis of the qualitative interviews mentioned earlier (please refer to Hippocampusproject.eu).

In the remaining part of this article, we will address how relaxation and the need to relax were specifically described by the participants, especially in the log material. We will also explore the relationship between yoga and relaxation, sleep, stress, and trauma based on the Norwegian qualitative data from the Hippocampus Project.

### 4.3. Yoga as recognition of the need for relaxation

The interviewees emphasized that practicing yoga facilitated their ability to relax and made them more aware of their need to relax. As we will demonstrate below, most interviewees found practicing yoga calming, relaxing, and helpful in counteracting stress.

A high school girl named Fay, who has ADHD and previous experience with meditation, expressed that yoga deepened her experience: “*It's the inner peace since I've definitely gotten stronger after doing yoga (...) yes, inner strength, mental strength, and yes, I am calmer.”* The sense of calmness was also emphasized by a girl in secondary school named Emile, who experiences anxiety. However, she mentioned that, for her to really enjoy the benefits of the relaxation part at the end of a yoga class, she needed a slow pace in the class:

*The minutes when I'm there, it seems to be helpful, but it helps the most when I'm calm and not super stressed. The breathing exercises and the mindfulness activities that we do works kind of fine, but the exercises [asanas] just make me feel that, if we do them in a slightly hasty way, then it only makes me more stressed*.

Thus, the importance of adjusting the yoga sequence to the needs of the individual has been demonstrated. In this case, the interviewee expressed that slow-paced yoga allowed for calmness during relaxing poses, while high-paced yoga did not.

A boy in secondary school named David, who has ADHD, reported that he found yoga helpful since it allowed him to remain relaxed rather than be stressed during exams: “*I manage not to be stressed if it was a test, for example. I just sit there relaxed. Because then you can think of something else… just like when you just do this with your body [sits still in a meditation pose, eyes closed].”* Another secondary school boy, Cole, shared his thoughts from his experience with Yoga Nidra, a relaxation exercise that invites calm alertness: “*You are aware of what is happening, but you don't pay attention to it. When we do Yoga Nidra, it feels like you're falling backward when you are actually not moving at all. It's not like bad weird, it's like (...) cozy and relaxing.”*

A Syrian girl named Barbara shared how yoga increased her sense of calmness and reduced her stress levels when she had to perform at school:

*(laughs) Erm, for example, in school too, when we need to do a presentation or something, then it will go well; you can do that. I do this [breathes in, lifts arms, and taps her fingers], and it becomes better. Erm, yes, and what I said about controlling myself and not being stressed; being calm, and yes, just having less stress*.

Idil, a girl from Somalia, expressed that she found yoga to be helpful, mostly for sleep and relaxation, and that she loved the sensation of focused breathing in yoga. She described it as follows: “*Breathe in slowly with the belly. Just the belly flows.”* When the interviewer inquired about what she experienced, “*And that relaxes your body?”*, she described further, “*Yes, my fingers, my feet, my whole body.”*

David illustrated how the sequencing of the yoga poses was relaxing to him: “*I also liked it when you go from plank, and then you sit down because then you can calm down in a way and breathe out.”* He found that the relaxation asana (Shavasana) changed his work mode at school: “*I manage to fall asleep almost every time, and then I can manage to work better afterward.”* It was quite common among the students to fall asleep during relaxation (Shavasana, lying down on one's back, is often done at the end of a yoga session), and the yoga group that David was part of even called Shavasana “the sleeping exercise.” “*You become kind of sleepy, and then, maybe feels like I'm smoothly closed off, and I just start all over again.”*

For some adolescents, an extensive focus on negative news and events can be stressful, as it might influence their everyday life:

*I've been thinking that in school and otherwise, we talk a lot about negative stuff. And then, it is better for us that we can manage to relax and feel that we are in the real world; you get kind of desensitized when you hear about a murder at school and things that have happened in the past. It is important to learn, but when there are very few positive things one talks about daily... I think that is something that influences mental health a lot. I felt that it helped a lot to just lie down, breathe deeply, and just feel that you are a part of your body* (Fay).

For this informant, it seemed that the experience of lying down and focusing on deep breathing allowed for a reconnection with the body and the ability to relax. Indeed, the body can be a barometer for feeling tensed or relaxed. For example, Hugir, a Kurdish refugee boy, expressed: “*Sometimes you are tired, and you go to yoga and breathe in and out. Your body feels very nice, so you relax and stuff.”* Like several others, this participant described how yogic breathing, that is, pranayama, provided good feelings and a sense of relaxation in the body.

Practicing yoga can also provide a sense of wellbeing. For example, Cole expressed that the breathing exercises in yoga class induced feelings of happiness and relaxation:

*They [those doing yoga] feel better after yoga because they are relaxed. Especially after, when we do those breathing exercises and lie on the floor and concentrate on our own breathing; then, everyone is happy, I think. We are more relaxed after yoga than the others [not doing yoga]*.

Furthermore, he explained why he felt that doing yoga was a positive experience:

*Yes, and at the end of the day, the school day, it is pleasant to lie down on the ground and just breathe. You don't think so much about what is going on around you (..) so you don't mind about other people, you are just with yourself. Maybe I don't get so influenced by stress anymore*.

It appears that yoga allowed this young boy to be with himself, pay less attention to his external environment and peers, and thus, become less susceptible to stress.

Fay found relaxation to be an essential exercise for young people to handle feelings: “*Teenagers today have a lot of problems by feelings being stirred; it can become a lot to handle, and then, many start skipping school. But when you manage to relax a bit, you don't feel the same stress.”* For this informant, relaxation was interpreted as a buffer against stress and even school absenteeism. She had learned a few meditation and breathing exercises before the yoga intervention but concluded that yoga gave her an extra release: “*In the yoga class, I think that the beginning helps with releasing some of the restlessness. With that, you can just move a bit and stretch, and all the muscles in your body become loose.”* She also found Yoga Nidra to be effective:

*I think it [Yoga Nidra] helped me. It was definitely my favorite part of the whole class: to just lie down and listen to those instructions that you go down through your head, shoulders… I liked it too; it's nice, and you get so calm by doing it. I got sleepy and tired! But when I left after the class ended, I got a bit more energy*.

### 4.4. Relaxation as expressed in the log material^14^

The logs displayed reflections on how yoga influenced the students involved in the Norwegian Hippocampus Yoga Interventions. These logs emphasized and supplemented the findings based on the qualitative interviews with disadvantaged youth. Approximately two-thirds of the logs emphasized enhanced relaxation and appreciation of relaxation. Below, we have shown some examples of statements with regard to relaxation. The first example is quoted from a girl attending a class of hairdressers:

“*I think that doing yoga gives the body relaxation and that also relaxation after a long day working in the standing position. It's lovely to have a class where we can relax and think about something other than work.”*

Another student at HS1 wrote this about yoga:

“*I think it has been good because it helps me relax. And it helps me gather the thoughts that I stress with on a daily base. Also, my body can relax physically and mentally.”*

One of the students in the introductory class of migrants at HS1 wrote:

“*I feel I get to relax in my whole body, mind, and thoughts.”*

A boy in secondary school (SS) seemed rather pleased to feel relaxed:

“*This was lovely. Now I'm awake and have a lot of energy. I just have one word: relaxed!”*

A student who disclosed having PTSD felt the relaxing effects of yoga:

“*Every time before yoga, I have always been stressed out about something. In addition, I'm never fully relaxed because of PTSD. After yoga, I feel that I am actually able to relax.”*

The teenagers also testified to the effect of sequencing, that is, using the body in various movements (asanas) first and then relaxing:

*It felt good to have some variation today. It was nice to start as before by focusing on the breath, and after some time, to go on to do some stretching to soften up the body in the middle of the school day. In the end, I almost fell asleep since I got so relaxed, and I could feel that this was something I needed*.

Moreover, some of the students noticed a lingering relaxing effect during the day, which was appreciated:

“*I felt so much more relaxed and calmer during the day, so it has been very good and useful. I liked having yoga Tuesdays, so it's too bad that this is the last time. But I'm very grateful that I got to be a part of this.”*

Some students reported that they clearly needed a timeout:

“*I am very tired and sleepy. So, in this class, I just want to relax. When we lied down on the mat, I tried to pay attention, but I was so tired that I fell asleep. This was good because I feel much better now, even if I am still a bit sleepy.”*

At the International School (IS), yoga nidra and relaxation exercises were immensely popular. Thus, the yoga teacher, who was also a teacher at IS, decided that they would have entire classes of relaxation. The students appreciated the fact that she considered their wishes:

“*We only had relaxation. It was great after a long day at school! This was my favorite lesson!”*

Generally, teenagers' need to relax is well known, but the respite from the constant evaluation was appreciated, along with a break from sitting in class all day:

“*Good to get away from school classes with all the sitting and work. It's good to log off, relax, and do some movements now and then. I fell asleep and was tired, but I needed it.”*

Furthermore, just a short break seemed to lead to perceptions of being refreshed:

“*Today, we started with noticing the body and stretching the joints. Very comfortable and slow session. We were supposed to just lie down and relax, and it feels like I've been sleeping for an hour. I feel very relaxed.”*

Accompanying the feeling of being relaxed was also an awareness of being awake and calm, as illustrated in the following two comments:

“*I was relaxed in my body and mind. I forgot about the stress. I became more awake but at the same time sleepy.”*

“*I like yoga a lot because I feel more awake and get a kind of inner calm. I feel quite relaxed. I feel my breath more than before we started. My legs are aching a bit.”*

As was demonstrated in both the interview data and the logs, feeling more relaxed could also make some of these young people more sleepy or more aware of being sleepy.

Teenagers are heavy users of mobile phones, which can be a source of connectedness and stress in their lives. Some of the refugee informants shared their impressions of being addicted to their phones:

“*I am comfortable with yoga. It's good for forgetting about the phone. I forgot about everything, and I don't think about anyone or anything.”*

“*I don't want to be addicted to my phone. I am addicted to healthy food.”*

“*I can sit without using my phone for a long while.”*

“*It's good to do yoga on your spare time; that way you don't have to use your phone.”*

Here, yoga is viewed as a way to reduce or regulate the informants' phone use and, thus, reduce tension. In the log material, especially the refugee informants described how practicing yoga made them more relaxed. Several of them mentioned that it contributed to a more relaxed body, breath, and mind. In addition, they pointed out how yoga influenced their mental state and fatigue level, providing them with a needed pause or redirection of focus:

“*I feel that I can relax in my whole body, mind, and thoughts.”*

“*It was good for the brain. The mind became calm.”*

“*I think it was very good for focusing the mind, and the mind becomes calm.”*

“*All negative thoughts are gone after yoga.”*

“*I feel very good after yoga: no stress, better focus, and relaxed.”*

“*Yoga helps me take a break or move away from the thoughts that disturb me. And I feel that my body is in good shape after yoga.”*

Thus, several of the refugees revealed that they felt better, calmer, and also more focused as they experienced fewer disturbing thoughts and reduced levels of stress. Moreover, relaxation allowed them to feel tired and sleepy.

### 4.5. Yogic relaxation and sleeping habits

Interview data suggested that yogic relaxation seemingly improved sleep patterns among the young participants and pointed to a newfound agency to regulate lifestyle habits, such as sleep. The awareness of being sleep-deprived seemed to allow the informants to get more sleep.

The need for sleep was especially cited by the young people with refugee backgrounds; for example, Ayla, one of the Syrian girls, mentioned, “*After yoga class, I just want to sleep; I don't feel like continuing school classes.”* She further described how a friend in the yoga sessions responded: “*When she [the yoga teacher] says ‘just relax and close your eyes and stuff, she sleeps.”* In addition, she found that practicing yoga changed her own sleep habits: “*After yoga, my sleep has improved.”*

Idil, the Somali girl, attempted to describe how her sleep had improved during the 8-week yoga intervention. We have included the dialogue with the interviewer to shed light on the probing into her experience:

*Idil: “I felt very relaxed. And used to get a very good night's sleep.”*

*Interviewer: “Did you do yoga at home then?”*

*Idil: “No, I would just try right away. Yes, I sit with myself and try to… I try to sleep well. Yes, because my body is tired. So, I just care about that I want to sleep.”*

“*Mmmhm… sleep is better yes, so it's good for my body (…) I have to try some more at home so that I can find peace, and it's a bit better for me.” (Mother Language Assistant's translation of Somali): “I notice that when I do yoga, I fall asleep faster whereas earlier, I was always staying up late.”*

This informant deliberately aimed toward using yoga to sleep and to listen to her body when she was tired rather than staying up late. When asked about how yoga had influenced her life, she responded as follows: “*I feel very relaxed in my body, and I'm able to relax and to go to bed when I need to.”*

Similarly, Javid from Afghanistan reported being able to completely relax and fall asleep in yoga class and to use some of what he has learned when going to bed at home:

“*Yes, it was very good for the body when I did it... but when we would finish the class, I was a bit tired. Yes, and I sleep very well at night and such.”*

*Interviewer: “Do you sleep better at home as well?”*

*Javid: “Yes, sometimes. Better at times and sometimes not so well.”*

*Interviewer: “Yes, and when you go to yoga, it was the last exercise when you lie down like this...?” [referring to the yoga pose in dialogue]*.

*Javid: “Yes, I was immediately asleep.”*

Javid explained that he used yoga to sleep better at home, and similar to the other interviewees with a refugee background, he seemed to find a safe space to relax and let go, with total relaxation prior to dozing off to sleep.

### 4.6. Yoga being beneficial for trauma, wellbeing, and connectedness

The refugee informants seemed to have a different level of unrest and challenges to their wellbeing since they had traumatic memories extending into their current situation. They were all in a special learning environment in an ordinary high school, as well as in the process of cultural integration and language learning.

Javid from Afghanistan was alone in Norway with no family and expressed that yoga was good for his body, mind, and sleep. Furthermore, he mentioned that it had positive effects on his stress levels: “*When I have a headache, then I try yoga, and it feels very good and cozy.”* He was clear on the effect he felt from focused, intentional breathing. “*Yes, and I breathe… the whole body breathes. The whole body breathes like we breathe.”* While he disclosed that yoga did not always make him feel better, he mostly had a better day after the yoga class: “*After yoga, it would feel like… at the same time good and not so good: sometimes good and sometimes not good. 50–50.”* When Javid was asked if yoga had influenced his life in any way, he responded enthusiastically: “*Yes, of course!”*

Ayla, one of the girls from Syria, was referring directly to the memories of war: “*Yes, we let go of all the things… the war and things like that. It's... you know, difficult, but we let go of negative things. Yes. We only think of the positive things.”* She reported being more selective of her thoughts: “*You don't have to focus on two things at a time. Yes, first I must focus on this, and then afterward, this. So, I organize everything in my head.”* She was fairly positive about the potential she saw as possible from having a regular yoga practice and genuinely appreciated what she had learned: “*If you continue doing yoga, you can learn everything; you can focus and let go of thoughts you don't need to think about. I'm lucky to have tried yoga.”*

Traumatic memories can distort the ability to concentrate and disturb current activities. The mother language assistant accompanying Hugir explained after a longer sequence in Arabic:

*MLA: “He could actually relax in the yoga class, especially when he closed his eyes and focused on his breath. He also noticed that he is clearer in his head afterward.”*

*Interviewer: “Could you explain more about getting a clear head? Could you elaborate?”*

*The MLA responds after another sequence in Arabic: “Like old thoughts and memories fade a bit. It's also a break from the teaching, and then they are focused because there is so much to learn. Then they can relax a bit and not make any effort.”*

Hugir summed up his experiences with yoga as follows: “*Yoga feels very nice in the body. Then I get clearer thoughts. And it is good for the mind and soul.”*

It also seemed as though practicing yoga together created an environment of psychological support and safety for the group of refugee students. Javid expressed that, even though yoga sometimes helped him sleep, his life situation as a refugee was evidently affecting him:

*MLA: “Sometimes, he doesn't sleep. Sometimes, he thinks a lot and cannot sleep (…) his family lives in Afghanistan; often he thinks of his family instead of sleeping.”*

*Interviewer: “Has yoga influenced how you feel inside?”*

*Javid: “I had a very good experience with the yoga course. I can use it in daily life.”*

Through the mother language assistant, he affirmed what he had mentioned earlier about how the ambiance of the yoga class made a difference: “*Yes, it's different to have a yoga class than other classes, because in yoga class, the focus is on being calm, and the whole group understands what you are saying. But, in other places, it's not the same.”* However, the need for a supportive environment was not important only for the refugee interviewees.

Norwegian teenagers Fay and Cole noticed how the yoga classes created a feeling of being connected in the class. Fay, for example, expressed that she did not socialize much with her classmates: “*But I see that they are more open in a way. They're not... it was a while when they weren't that negative, and I appreciated that. Always when we were done, we were happy and just talked to each other and were very open.”* Similarly, Cole noticed an openness that he ascribed to the whole setting: “*It's not just them who look like they are happy; it's also [makes a broad movement with his hands]... the environment really. Yes, you are not really sure what you've experienced, but you can feel it. I just feel better. I feel happy and relaxed*.” Yoga seemed to have contributed to a group dynamic where kindness, support, and feeling safe were experienced by the refugees and Norwegian informants.

A few of the young participants noted that even the setting and ambiance of the yoga class had a certain effect, perhaps reflecting an anticipation of being allowed to relax: “*Now I feel fine. I felt calm when I entered the room; it was silent and comfortable. I'm a bit tired and sleepy. I'm looking forward to the sleeping exercise at the end. I look forward to yoga.”* One student with a refugee background had trouble sleeping and asked if the yoga teacher could help him sleep better. The teacher made a body scan recording for him with instructions to relax the whole body. This informant reported managing to sleep better after listening to the recording at night prior to going to sleep. The body scan recording became popular among the entire class of newly arrived refugees at HS2 (10 students). Yoga seemed to be especially beneficial for the refugee participants who emphasized relaxation, rest, and a reduction in negative thoughts and memories.

### 4.7. Mixed feelings about practicing yoga

Even though most of the participants appreciated yoga and reported increased relaxation and improved sleep, in the log data, there were also examples of more negative experiences with yoga. Some of the logs refer to variations in the perception of yoga, and even though yoga provides relaxation, some logs also describe mixed feelings. For example, one student appreciated the relaxation but also experienced dizziness:

“*I liked the last exercise very much; it was something about relaxation [Shavasana]. I also liked the child's pose, and I think it was a nice exercise when someone lay over me the last time when I was in the child's pose. I got a bit dizzy from the first few poses [Sun Salutation], but it went well.”*

Another student at the IS shared feeling uncomfortable and tense:

“*I felt uncomfortable throughout the yoga session. I felt slightly stressed, and my body felt tense. At the beginning of the session, I had trouble being calm. Later in the session, I could feel that I had really used my muscles.”*

There were also log statements describing headaches and pain from doing the yoga asanas:

“*I had a headache during the yoga session.”*

“*I felt pain. The worst one is the downward dog pose [Meru Asana].”*

“*I'm even more tired now. My back hurts.”*

Some of the informants, such as a few students in the introductory class of migrants at HS1, disliked yoga:

“*I think yoga is boring because I don't like quiet activities.”*

“*Yoga is good for health, but I don't like it.”*

Similarly, a boy at IS was disappointed by yoga and also found it boring:

“*There really isn't any change in my body. It was boring. The meditation was annoying, and the only result from that is sleepiness.”*

Others described having mixed feelings about their yoga experiences:

“*I didn't like yoga! It's boring! You're not allowed to laugh.”*

However, later the same person wrote:

“*I think it was quite lovely to do yoga, but it's a bit boring.”*

Their feelings seemed contradictory, as one boy expressed:

“*I'm fine, but I have a headache. I think we should have yoga more often. I think yoga is boring.”*

Another boy also mentioned having such mixed expectations and experiences:

“*I'm sleepy… not grumpy but not quite happy. I'm not looking forward to it much, but I think it will be good to stretch.”*

After class, the same person wrote about body pain and relaxation as follows:

“*My chest hurts. Relaxed. Headache. It was all right. Rested.”*

The mixed feeling could also be related to the expectations that several of them expressed about yoga's ability to reduce stress and enhance their performances. One boy explained as follows:

“*I expect to relax. I really hope yoga helps the brain.”*

At the end of the yoga intervention, the same boy expressed:

“*I want to say that it has been totally ordinary to have done yoga. Nothing changes. But you get tired.”*

The latter statement can be interpreted as the informant being disappointed by unfulfilled expectations. It is possible that a few of these mixed feelings about yoga, which were mainly mentioned by some of the boys, were a way of distancing themselves from yoga, which some of them also described as “sissy” or “gay.”

## 5. Discussion: is relaxation the main yogic contribution?

The purpose of the Norwegian part of the Hippocampus Project was to gain insight into the experiences of young people in Norway who were exposed to the project's yoga intervention. As discussed at the beginning of this study, discourses related to adolescents are often focused on the challenges and what is missing. The neoliberal political hegemony emphasizes competition and the need to succeed (Krokstad et al., 2022), thus inducing a fear of failure rather than hope. Generally, as Guyer et al. (2016) pointed out, adolescence can be a stressful period of life. Teenagers undergo major neurological and hormonal changes in addition to social and cultural changes (Martinus, 2018). The educational demands and expectations of future competence and employment add to their already pressured situation. The study participants seemed to experience pressure and stress related to school, social relations, and social media.

Our main research question was the following: What was the experience of the young students at the Hippocampus Yoga Intervention offered at Norwegian schools? One of the main findings is that the participants appreciated how yoga allowed them to relax. The logs from 133 individuals confirmed the results of the nine individual interviews about how yoga encouraged restorative states of relaxation and sleep and developed healthy ways to cope with stress and PTSD. Practicing yoga made them calmer and more aware of their need to relax. Yogic breathing, or pranayama, was highlighted by several informants as being beneficial for slowing down and being able to unwind. In addition to highlighting yogic relaxation, one of our study's strengths was that we combined qualitative interviews with the log material. These two data sources both confirmed and supplemented each other well, especially regarding yogic relaxation. Another strength in the study design pertains to the yoga interventions as an everyday routine in life at the schools, in the classes, and in the groups in the schools where we recruited our informants.

We were also interested in the following question: How did the students feel the yoga course impacted their mental health and wellbeing, perception of stress, and sleep patterns? Our findings reveal that young people considered yoga a supportive tool that could motivate them to make lifestyle changes, such as accepting and recognizing the need for rest and relaxation, as well as understanding the value of appropriate sleep habits. The opportunity to relax seemed to empower them and improve their ability to self-regulate, which is also sleep related. The young participants mentioned that yoga facilitated their counteraction and their ability to cope with stress and PTSD-based experiences. Recognizing the basic need to relax and wind down during a school day and having the tools, time, and permission to fulfill that need is crucial in preventive health care. Being calm and relaxed can be a way to create a safer environment, thereby enhancing relational health, that is, how people relate interpersonally as well as intrapersonally (cf. Kishida et al., 2018). All these factors positively contribute to young people's mental health and wellbeing.

Being offered opportunities to relax deeply also impacted the informants' awareness of their need to sleep. For some, the relaxation technique at the end (Shavasana) became a “sleeping hour.” For others, the newfound ability to relax improved their sleeping patterns, motivating them to go to bed when they felt tired/sleepy. Our data displayed numerous examples of how yoga was especially beneficial for many of the refugees by creating a safe environment and lowering their often trauma-related guard. For instance, the requested creation of a 20-min audio file with the body scan meditation improved the sleep of at least one refugee' and was appreciated by the other students who also obtained access to the tape. Such a simple yogic tool could provide support and feelings of safety for newly arrived refugees and would be an effective, low-cost measure for improved sleep quality for people suffering from insomnia due to traumatic stress.

Yoga seemed to be especially beneficial for the refugee participants who emphasized relaxation, rest, and a reduction in negative thoughts and memories. PTSD was common among the refugee informants, with reoccurring memories of war and worrying about family remaining in the home country adding to the distress, which means being in an overly alert mind–body state, with poor sleep quality and difficulty concentrating. This group also mentioned their addiction to mobile phones, a behavior that can make it even more difficult to sleep. However, adequate rest and sleep are essential for the brain and the body in all stages of life, especially for young people, given the intensive changes in the brain and the body (Khalsa and Butzer, 2016; Saksvik-Lehouillier et al., 2020). The participants seemed to be sleep-deprived due to stress and tension, especially the young refugees with traumatic memories. Emotional regulation, cognitive function, and memory are all highly influenced by our quality of sleep and relaxation (Tarokh et al., 2016).

Yoga offers a practical way of working with the body, breathing, and having a focused mind before going into a relaxation mode; thus, excess energy and activation are released before transitioning into a rest period. As indicated, some of the yogic asanas and pranayamas downregulate the sympathetic mode of the autonomic nervous system and allow the activation of the parasympathetic mode (Vasanthan et al., 2017). This facilitates rest, recovery, and mental, emotional, and physical restoration. This shift of mode allows us to digest our food and accept our experiences instead of constantly operating in the alert sympathetic mode that is constantly preparing us for fight, flight, or freeze responses. A high level of excess energy and activation is common in many young people as they experience everyday stress related to school, social media, and the related lack of sleep. Research has found that yogic relaxation practices, such as Shavasana, can counteract stress and sleep deprivation. Raghul et al. (2018, p. 1) concluded, “Such conscious relaxation may be able to correct an imbalance of the autonomic nervous system by enhancing parasympathetic tone and reducing sympathetic overactivity.” Similarly, Vasanthan et al. (2017) found that relaxing asanas and pranayamas can enhance parasympathetic activity and decrease sympathetic activity (cf. Frank et al., 2020).

It is widely recognized that appropriate exercise is an important component of stress reduction, sleep, and emotion regulation. Yoga provides several additional factors to the movement of the body through asanas that demand focus and balance. Furthermore, the focus on coordinating the breath with movement and a whole range of pranayamas (breath control techniques) can be beneficial, as the participants in our study highlighted. Breath is the most accessible function in our bodies that can influence the vagus nerve by lowering the rhythm of the breath and focusing on extending the exhale. Thus, the increased vagal tone (which means that the vagus nerve functions more optimally) supports the body in essential functions, such as digestion and the subconscious aspects of breathing, thereby giving vital organs the resources and ability to function well (Rosenberg, 2017). The ventral vagus nerve is activated when we feel safe, and continued activation of this part of the nerve calms us down, which is a central state for thinking clearly, integrating learning, and regulating emotions.

The asana practice involving stretching large muscle groups in the legs and the torso releases stress and gives the brain signals that one is feeling safe and calm, leading to the brain evoking more of that experience of safety with continued practice and stimulating an upward spiral toward a less alert nervous system (as described by Heather Mason at the Trauma Super Conference in April 2022, https://traumasuperconference.com/). Our data suggested that the participants who had experienced war trauma tended to embrace yoga as an activity that is beneficial for their sleep, relaxation, and ability to calm down. This is probably experienced through stimulation of the safety response of the ventral vagus nerve by stretching and breathing in an environment with an ambiance of safety. Both the refugees and the other high school and secondary school participants seemed to find a certain sense of openness and friendliness to characterize the yoga class. As described by Rosenberg (2017), the activation of the ventral vagus nerve enables us to communicate better and bond well with others. This ability of yoga to enhance social connectedness among participants was also proposed by Kishida et al. (2018).

The amygdala, the center in our brain popularly referred to as the alarm system alerting us to real and imagined danger, is triggered by any aspect, varying from social comparison and imagined threats to real dangers (Goleman and Davidson, 2017). Constant checking of social media, demands to stay on top of things, keeping things under control, and trying to be perfect according to the demands of modern life activate the amygdala, sending stress response signals as well as creating a reaction of neurotransmitters and hormones to flow in our body and stimulate actions as if we are in danger. The yoga asanas stimulate the parasympathetic nervous system and demand our focus due to the level of difficulty and complexity required for balancing skills and motor activation. Physical asanas are typically followed by guided relaxation and then Shavasana (also called the Corpse Pose or Dead Man Pose). The closing of a yoga sequence with a relaxation technique deepens the activation of the parasympathetic nervous system and encourages our system to linger in this mode for longer spans of time (cf. Gitananda, 1981).

Our findings suggest that most of the young people in our study appreciated practicing yoga, and these sessions allowed them to relax and induce sleep. Several of the participants expressed that they felt calm and reenergized after the yoga sessions. A sense of becoming calm, peaceful, and relaxed when doing yoga was also highlighted in a recent article by Kishida et al. (2018). However, based on our research, it is imperative to mention that yoga did not appeal to all the participants in our study. While the majority of the participants appreciated yoga as being relaxing, some also associated its practice with boredom, discomfort, dizziness, headaches, and muscle pain. It is possible that these described discomforts were related to traumas, as some of the informants describing such negative bodily experiences had a refugee background. For future yoga interventions, it may be helpful to place more emphasis on yogic breathing techniques (pranayama) and to create safe and supportive environments that encourage comfort and mutual trust among the group practicing yoga. These approaches can be effective in addressing both general stress and PTSD (Maddux et al., 2018).

It is not a given that yoga will have any immediate effect. Even though many informants experience a sense of calmness and deeper relaxation than usual, it is common to experience stress more intensely when one tries to relax or has time to relax. Some participants mentioned that practicing yoga did not meet their expectations for change or health improvement, while other participants expressed dissatisfaction with the strict adherence to political correctness in the yoga sessions, which included a ban on laughter. Others expressed that, even though yoga was healthy, they did not appreciate it. It was perceived as being too quiet for some of the log writers. In particular, some of the boys had mixed feelings about it. In addition, a few of the male informants said that practicing yoga was too feminine or “gay” and thus felt the need to distance themselves from yoga. Such a perception of yoga as a feminine activity, along with the masculine ideals in society, have been identified as a few barriers for men doing yoga (Cagas et al., 2021). It may play a role for our findings that yoga is dominated by women in Norway, as in many Western countries, and that our yoga teachers in the Norwegian Hippocampus Intervention were all women.

The interviews and log data point toward a newfound agency to regulate behavior and habits in a way that meets the need for relaxation and getting enough sleep. This new agency can be linked to increased self-awareness and empowerment to choose a lifestyle based on the changes in attitudes toward resting and getting adequate sleep. The interviewees' statements about their dependency on mobile phones could be an example of such increased awareness. Young people's constant use of and availability through social media can be a stressor in their lives (see Rice et al., 2018). In contrast, yoga seemed to make the participants more aware of their lifestyle, media habits, and use of time. Modern yoga, including awareness-building meditation techniques, has mostly been embraced as a stress-reduction tool. One could claim that yoga and mindfulness have become valuable components of the stress management industry. Stress reduction through yoga has also been the focus of research (Riley and Park, 2015; Dariotis et al., 2016; Park et al., 2021), which we find extremely valuable. However, in this study, we have focused on the relaxation aspect emphasized by the participants and highlighted relaxation as an effective practice to counteract stress and facilitate health improvement.

Yoga pertains to achieving balance in life (see Bhavanani, 2017). If young people become aware of the natural rhythm of rest and activity needed to be productive and alert without becoming tense and exhausted, they will be better able to withstand stress (cf. Bhavanani, 2009). This could enhance their ability to create a balance between learning and playing, which would have several benefits for mental health and wellbeing. This, in turn, is vital for learning and memorizing, having a supportive psychosocial environment, and connecting with and cooperating with classmates. As we argued in a previous article, yoga is a useful tool and practice for wellbeing that schools need to welcome as it could influence their students' life skills and help them better deal with their emotions (Hagen et al., 2021).

The study participants showed increased awareness of the importance of relaxation, stress reduction, and improved sleep, even though they had practiced yoga for only 8 weeks. This is in line with what we described earlier: practicing yoga is about awareness-raising: increasing one's awareness about the body, mind, and feelings, and about being aware or not (meta-awareness) (Gitananda, 1981). If yoga can enable young people to relax better and become more mindful of their need to relax and sleep, then introducing yoga can be essential for their mental health and wellbeing. According to Bhavanani (2016, p. 2):

*Relaxation is a central element in yoga, as it is the body's own way of recharging its cells and helps to ease physical, emotional, and mental tensions. We can facilitate our own healing when we are relaxed. We often unintentionally retard our inherent healing mechanisms when we are tense and uptight* (Bhavanani, 2016, p. 2).

The increased awareness often experienced at different levels when practicing yoga promotes young people's sense of empowerment and can support healthy lifestyle choices related to rest, sleep, and regulating the use of social media, as well as relieving psychological tension and trauma-related challenges.

Yoga needs to be taught as a holistic approach to promoting a healthy balance of action and relaxation, similar to the way it was attempted in the Hippocampus Project. Such an integrated version of yoga, including ethical reflections, physical postures, breath regulation, and meditation, was proposed by Kishida et al. (2018). The authors claimed that, “Indeed, this upward spiral generated by enhanced autonomic flexibility may be pertinent for the stress-relieving effects of yoga and for optimal self-regulation (e.g., attaining relaxation may restore cognitive resources which are necessary for successful regulation of behavior)” (Kishida et al., 2018, p. 220). Yoga can provide relaxation and act as a means of counteracting and coping with stress, thus facilitating improved wellbeing and public health. The strength of our study was exploring the benefits of yogic relaxation, both in the way this was articulated in the interviews and emphasized in the logs. The yogic approach is closely related to the salutogenic model, which underlines the factors promoting health. Yoga needs to be promoted as a way of life to achieve this. Therefore, it is well-suited to be a part of complementary or integrative medicine and therapy, thus empowering young practitioners to maintain a healthy equilibrium of activity and relaxation.

## 6. Conclusion

The contribution of yoga to relaxation and increased awareness of relaxation were the major findings of this study. Relaxation and awareness seemed to counteract their levels of stress. Moreover, increased relaxation also induced sleep and increased awareness of the need to sleep. The importance of relaxation and rest was particularly emphasized for participants with a refugee background, mainly due to the traumatic events they experienced that made them more vulnerable to stress. As a result, they found the yoga sessions beneficial in terms of promoting relaxation and improving sleep.

The increased awareness that these young people developed by practicing yoga for a limited period can inspire them to make healthier lifestyle choices. Additionally, they gained concrete yogic tools and skills to facilitate relaxation and have additional balance in life. Adequate relaxation can improve people's mental health by increasing their self-healing capacity and wellbeing, contributing to teenagers' positive development and learning ability.

The contribution of yoga to young people's ability to relax and awareness of relaxation is worthy of further exploration. This might supplement the literature on how yoga can reduce stress. Awareness concerning relaxation and skills for relaxing could potentially be one of the most important contributions of yoga to the young generation's mental health and wellbeing. Increased understanding about relaxation, sleep, and stress levels through yoga may also support young people's life mastery, personal growth, and ability to thrive at school and in modern society.

## 7. Limitations

This study has certain limitations. Since the Norwegian quantitative results were not statistically significant (due to attrition), we could not benefit from the mixed-method strategy in the European Hippocampus Project. Despite this, our qualitative data were interesting and could deepen the tendencies we noticed in the Norwegian quantitative data.

There were potential limitations due to the way we selected the interviewees. The teachers and yoga teachers played a role in selecting interviewees with mental health problems and/or challenges. Furthermore, the students had a chance to volunteer for the interviews. This could have led to certain biases. Nevertheless, all the participants fulfilled the criteria of being disadvantaged, which was an important criterion for selection in the Hippocampus Project.

The interviewer had experience with yoga as well as with qualitative interviews. Having prior experience with yoga could be considered a potential interviewer bias. However, knowledge of yoga was a resource that enabled the interviewer to tune into what the informants were saying and facilitate deeper probing. Using the joint Hippocampus interview guide as the starting point added to the transparency of the process.

In hindsight, we realize that we could have paid more attention to templates for interventions and criteria for reporting qualitative work similar to that recommended by Tong et al. (2007) and Hoffmann et al. (2014). In addition, higher involvement of the key stakeholders, such as students and teachers, in the intervention program's decision-making process from inception would have been beneficial, as suggested by Dariotis et al. (2016).

## Data availability statement

The datasets presented in this article are not readily available because in Norwegian. Requests to access the datasets should be directed to ingunn.hagen@ntnu.no.

## Ethics statement

The studies involving human participants were reviewed and approved by NDS.no. Written informed consent to participate in this study was provided by the participants' legal guardian/next of kin if they were under 16 years, and by themselves if they were over 16 years old.

## Author contributions

IH contributed to the design and planning of the Hippocampus Research Project and wrote the draft of this article. SS was mainly responsible for data collection and analysis of the qualitative interviews and logs, with input from IH and UN. She also wrote a summary report of the findings. SS contributed to the revision of the article. All authors attended the cooperation workshops for the Hippocampus Project, thus laying the foundation for our Norwegian Research Project.

## Footnotes

^3^ https://oda.oslomet.no/oda-xmlui/handle/20.500.12199/3480

^4^The Hippocampus Project involved Norway, Belgium, Italy, Spain, and the UK and was financed by the EU's Erasmus+-programme.

^5^See also https://www.ungdata.no/stress-press-og-psykiske-plager-blant-unge/.

^6^This is taken from the Norwegian Public Health Report 2018 (see https://www.fhi.no/nettpub/hin/grupper/psykisk-helse-hos-barn-og-unge/).

^7^Please refer to https://integralyogamagazine.org/stress-relaxation-yogic-approach/.

^8^Please refer to https://www.psychologytoday.com/basics/stress.

^9^In the yogic vocabulary, tension is referred to as Spanda and non-tension as Nispanda, and when practicing yoga one goes back and forth between Spanda and Nispanda (see Gitananda, 1981).

^10^The Hippocampus Project lasted 26 months and was funded by EU's Erasmus+ program (see Hippocampusproject.eu). The yoga interventions in schools reached over 750 young people and their teachers in all the five countries involved in the project.

^11^A parallel development of the instructional app Yuva Yoga based on the Hippocampus Yoga Intervention was ongoing. The app was intended to be a resource to stimulate further practice of yoga. However, as its completion was delayed, it could not be used in the intervention (please refer to García-Holgado et al., 2019).

^12^The analysis was validated by Martin Inge Standal who performed a thematic analysis of the same data using NVivo12.

^13^*The citations from the interviews and logs are all in Italics in the text*.

^14^Since many of the statements in the logs were short, we have chosen to present some of them as lists to display the variation in describing the same or a similar theme. This differs from our presentation of the interview statements.

## In memoriam

This article is dedicated to our dear friend and colleague, Professor Usha Sidana Nayar, who passed away in 2021. She was part of the Norwegian Hippocampus team and has also been a source of inspiration for several years.

## Appendix

**The Hippocampus Project interview guide: student interviews**

1. How was your experience with the yoga program? What do you think about the yoga program?
- Attention to yoga as a whole and specific exercises/eight limbs, sequence exercises.
2. How has yoga affected your life? Have you noticed any changes in your life since you started practicing yoga?
- What about your relationship with family, friends, and hobbies?
- School life: Is there anything different about being at school? Has something changed in how you learn new things?
- How does yoga affect your emotions?
- Has something changed in terms of what you think about yourself? What feelings and thoughts do you have about yourself? How you feel about yourself?
- Are there any changes in physical health or sleep?
- Is there anything else you know or think has changed?
3. Can you describe any changes you have noticed about the other students who have been taught yoga at school?
- What about the other students you practice yoga with in the yoga class? Have you noticed anything that has changed/is different about them?
- Can you describe something that is different about them after they started yoga?
4. If you could decide, what would you change about the course you have attended?
- The positions, relaxation exercises, or breathing techniques?
- What were your favorite activities in yoga class? What did you like best?
- Is there anything you learned in the yoga course that you want to carry forward in life?
5. Would you recommend yoga to other people?
- What would you say about yoga if you had to recommend it to someone? In what way would you convince others to do yoga?
- Do you want to continue with yoga yourself? How?
- How was your experience with the YuvaYoga App?
6. What other experiences related to yoga would you like to share with us?
- Is there anything else we have not asked about that you would like to mention?
